# Accessing Dietary Effects on the Rumen Microbiome: Different Sequencing Methods Tell Different Stories

**DOI:** 10.3390/vetsci8070138

**Published:** 2021-07-19

**Authors:** Mi Zhou, Eóin O’Hara, Shaoxun Tang, Yanhong Chen, Matthew E. Walpole, Paweł Górka, Gregory B. Penner, Le Luo Guan

**Affiliations:** 1 Department of Agricultural, Food and Nutritional Science, University of Alberta, Edmonton, AB T6G 2P5 Canada; eoin@ualberta.ca (E.O.); yanhong@ualberta.ca (Y.C.); lguan@ualberta.ca (L.L.G.); 2 Key Laboratory for Agro-Ecological Processes in Subtropical Region, Institute of Subtropical Agriculture, The Chinese Academy of Sciences, Changsha 410125, China; shaoxuntang@163.com; 3 Dairysmart and Beefsmart Consulting, Saskatoon, SK S0K 3R0, Canada; matthew@dairysmartnutrition.com; 4 Department of Animal Nutrition and Biotechnology, and Fisheries, University of Agriculture in Krakow, al. Mickiewicza 24/28, 30-059 Krakow, Poland; p.gorka@ur.krakow.pl; 5 Department of Animal and Poultry Science, University of Saskatchewan, Saskatoon, SK S7N 5A2, Canada; greg.penner@usask.ca

**Keywords:** bull cattle, rumen, amplicon-sequencing, shotgun-sequencing, diets

## Abstract

The current study employed both amplicon and shotgun sequencing to examine and compare the rumen microbiome in Angus bulls fed with either a backgrounding diet (BCK) or finishing diet (HG), to assess if both methods produce comparable results. Rumen digesta samples from 16 bulls were subjected for microbial profiling. Distinctive microbial profiles were revealed by the two methods, indicating that choice of sequencing approach may be a critical facet in studies of the rumen microbiome. Shotgun-sequencing identified the presence of 303 bacterial genera and 171 archaeal species, several of which exhibited differential abundance. Amplicon-sequencing identified 48 bacterial genera, 4 archaeal species, and 9 protozoal species. Among them, 20 bacterial genera and 5 protozoal species were differentially abundant between the two diets. Overall, amplicon-sequencing showed a more drastic diet-derived effect on the ruminal microbial profile compared to shotgun-sequencing. While both methods detected dietary differences at various taxonomic levels, few consistent patterns were evident. Opposite results were seen for the phyla Firmicutes and Bacteroidetes, and the genus *Selenomonas*. This study showcases the importance of sequencing platform choice and suggests a need for integrative methods that allow robust comparisons of microbial data drawn from various omic approaches, allowing for comprehensive comparisons across studies.

## 1. Introduction

The rumen microbiome plays an essential role in feed digestion, supplying volatile fatty acids (VFAs), protein, and other nutrients to the host for growth and development. The relationship between this microbiome and valuable production traits like feed efficiency [1,2,3] and milk quality [4] has resulted in many studies investigating rumen microbial diversity, with a view to potentially manipulating the rumen microbiome to improve host performance. 

Studies evaluating rumen microbial composition typically use one of two major sequencing approaches: (i) marker-based amplicon sequencing [5], or (ii) metagenomic shotgun sequencing [6,7]. Amplicon sequencing involves the sequencing of phylogenetically conserved marker genes (16S rRNA gene for prokaryotes and the 18S rRNA gene/ITS region for eukaryotes), and assignment of microbial taxonomy via alignment to dedicated databases (e.g., Greengenes and SILVA) [8,9]. In metagenomic studies, taxonomy can by assigned as above using extracted rRNA reads, or as is now more usual, via alignment to partial/fully assembled microbial genomes using tools like as MG-RAST [10] and Kraken [11] with or without contig assembly. 

Many factors influence the composition and function of the rumen microbiome, including diet, age, and host genetics [5,12]. However, studies have also revealed the existence of a core group of microbial taxa, present across species, diets, and age categories. *Bacteroidetes* and *Firmicutes* are typically the two major bacterial phyla [13], *Methanobrevibacter* is the main archaeal genus [5,14], and *Entodinium* is the predominant protozoan [15] within the rumen microbiome. Knowledge of the rumen microbiota in beef cattle is largely drawn from studies in steers, with only limited knowledge of rumen microbial composition in bulls [3] There is a well-defined relationship between host nutrition and fertility [16], but it is unknown if this is associated with microbial composition or function in the rumen.

Given the distinctive approaches to taxonomic assignment using amplicon and metagenomic sequencing, it is of interest to evaluate if they give comparable results, as this would allow data generated using different approaches to be more reliably compared. Given the current focus on dietary manipulation of the rumen microorganisms to improve animal performance, it is particularly important to be able to identify major shifts in microbial composition across diets. Moreover, the paucity of data available concerning rumen microbial composition in beef bulls limits our understanding of the interaction between fertility and nutrition. Therefore, to test this, the present study assessed differences in rumen microbial composition in intact Angus bulls fed either medium-energy or high-energy diets, using both metagenomic shotgun sequencing and amplicon sequencing of 16S and 18S rRNA genes. 

## 2. Materials and Methods

### 2.1. Experimental Protocol

Sixteen bulls raised at the Beef Research and Teaching Unit at the University of Saskatchewan were blocked by body weight (BW) and randomly assigned to one of two dietary treatments: medium-grain backgrounding (BCK, n = 8) or high grain (HG, n = 8). Animals were adapted to their diets for 30 days prior to the experimental period, and detailed dietary composition is given in Table 1. BW at farm arrival, BW at the beginning of the experiment, BW at the end of the experiment, and dry matter intake (DMI) are provided in Table 2. Samples of rumen content were collected from each animal following slaughter at the conclusion of the experimental period. A 50-mL aliquot was collected for microbial profiling, immediately frozen on dry ice, and subsequently stored at −80 °C awaiting molecular analysis. 

### 2.2. Genomic DNA Isolation from Rumen Content

Prior to DNA isolation, approximately 500 mg of frozen rumen content was thawed on ice. Total DNA was extracted using the modified repeated bead-beating with column purification (RBB + C) method, as previously described [1]. Briefly, microbial cell membranes were disrupted via three rounds of bead beating in the presence of lysis buffer. Following centrifugation, the filtrate was then subjected to DNA isolation using the QIamp Fast DNA Stool Mini Kit (Qiagen, Valencia, CA, USA), following the manufacturer’s instructions. DNA quantity and quality were verified via two consecutive readings on a Nanodrop1000 spectrometer (Agilent, Santa Clara, CA, USA), and DNA was stored at −20 °C pending library construction. 

### 2.3. Amplicon Library Preparation

A 50-ng aliquot of isolated DNA was used as the template for microbial 16S/18S rRNA gene amplification, using primers optimized for the rumen ecosystem [5]. The V1-V3 fragment of the bacterial 16S rRNA gene was amplified using the primers 

Ba9F (5′-GAGTTTGATCMTGGCTCAG-3′)/Ba515Rmod1 (5′-CCGCGGCKGCTGGCAC-3′); the archaeal V6-V8 16S rRNA gene fragment was amplified with primers Ar915aF (5′-AGGAATTGGCGGGGGAGCAC-3′)/Ar1386R (5′-GCGGTGTGTGCAAGGAGC-3′); and protozoal 18S rRNA gene fragment was amplified with primers RP841F (5′-GACTAGGGATTGGARTGG-3′)/Reg1302R (5′-AATTGCAAAGATCTATCCC-3′). Amplification conditions for each primer pair are listed in Supplementary File S1. Amplicons were purified using the QIAquick Gel Extraction Kit, according to the manufacturer’s instructions. Purified amplicons were subjected to 454 Pyrosequencing at a commercial laboratory (Genome Quebec, McGill University, Montreal, QC, Canada). 

### 2.4. Metagenomic Library Preparation

For each sample, 1 μg of the genomic DNA was randomly sheared using Covaris S2 (Covaris, Inc., Massachusetts, MA USA). Whole metagenome shotgun DNA libraries were constructed using the Illumina TruSeq PCR-Free Library Preparation Kit (Illumina, Inc., San Diego, CA, USA), following the manufacturer’s instructions. Briefly, the DNA fragments were subjected to end repair to blunt the overhangs. Following this, a 350 bp library insert size was selected using different ratios of AMPure XP purification beads (Beckman Coulter, Mississauga, ON, Canada). Sequencing adapters and primers were ligated into each end. Library quality was assessed using an Agilent 2200 TapeStation (Agilent Technologies, Inc., Santa Clara, CA, USA) and quantified with a Qubit fluorometer using a Qubit dsDNA HS Assay Kit (Invitrogen, Carlsbad, CA, USA). Finally, the sequencing was performed using the Illumina Hiseq2000 PE 100 bp platform at the McGill University and Genome Quebec Innovation Centre (Montreal, QC, Canada). 

### 2.5. Bioinformatic Analysis

Raw sequences were quality assessed using the FastQC program. Short and poor-quality reads were removed using the BBTools suite [17], with a Phred quality threshold of 20. Taxonomic assignment of amplicon sequences was performed using the Quantitative Insights Into Molecular Ecology (QIIME) wrapper [18]. Operational Taxonomic Unit (OTU) identification was carried out using the open reference picking method implemented in QIIME. First, sequences were clustered into OTUs based on 97% similarity cutoff. A representative sequence from each OTU was then aligned to the SILVA database v.128 (16S for bacteria and archaea, 18S for protozoa). Taxonomic classification for each of the OTUs was performed using UCLUST [19]. Rarefaction analysis was performed in QIIME to assess sequencing depth, and the BIOM file produced in QIIME was exported for downstream analysis. 

Following sequencing and quality control as described above, metagenomic shotgun sequences were assembled into contigs using the MEGAHIT tool [20]. In-house perl scripts were used to retrieve all complete bacterial, archaeal, and protozoan genomes from the NCBI RefSeq database (November 2017) to build a custom Kraken database. Microbial classification was performed in Kraken (v1) by assigning assembled contigs to the LCA in the custom database. Ranked taxonomic files were then exported for downstream analysis. 

### 2.6. Statistical Analyses 

Alpha and beta diversity analyses were performed in R using the Phyloseq package [21]. Comparative taxonomic analysis was performed at the phylum, family, and genus levels. Only taxa represented by >0.05% of sequencing reads in more than 4 animals/diet were kept for downstream analyses for both shotgun and amplicon data. All statistical analysis was performed in R (http://www.R-project.org/, accessed on 20 March 2021) and SAS (v9.2). Differences in microbial community structure were assessed using ANOSIM in R [22] with the following standards: 0.5 < ANOSIM R < 0.75 implies different profiles; 0.25 < ANOSIM R < 0.5 implies different profiles with some overlap; 0.1 < ANOSIM R < 0.25 implies highly similar profiles. Differential abundance analyses across diets were performed using Kruskal–Wallis within R. Significance was declared at a Benjamini–Hochberg adjusted *p*-value < 0.05. Regression analyses of the identified taxa between the two methods were performed using PROC REG in SAS (9.3). Significance was determined at *p* < 0.05, and trends were determined at 0.05 ≤ *p* < 0.10. 

## 3. Results and Discussion

The current study represents the first survey of rumen microbial composition and its response to diet in adult Angus bulls. Two commonly used sequencing approaches were used, marker-based amplicon sequencing [5] and metagenomic shotgun sequencing [6,7] to assess whether both would generate comparable results. 

### 3.1. Microbial Profiles Generated Using Metagenomic Shotgun Sequencing

Metagenomic shotgun sequencing of rumen content generated a total of 24,185,977 ± 4,226,137 sequences per sample, which were assembled into an average of 287,858 ± 104,326 contigs. Of these, 124,741 ± 45,825 were assigned to bacteria and 8608 ± 3602 were assigned to archaea (Table 3).

Detailed taxonomy assignment data are provided in File S2. It is surprising that for each sample, only 251 ± 112 contigs (<0.01% of the total contigs) were assigned to the unclassified phylotypes of Eukaryota, and none were assigned to the protozoal taxa.

**Figure 1:**
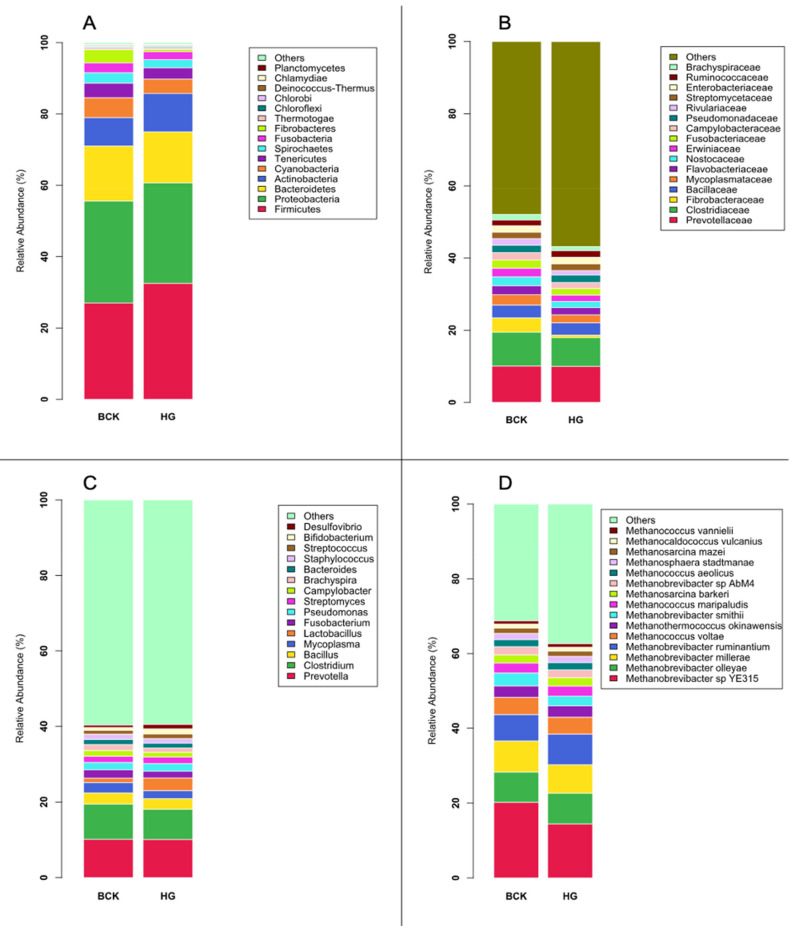
Microbial profiles at different phylogeny levels observed by shotgun-seq. (**A**) Bacterial phyla; (**B**) Bacterial families; (**C**) Bacterial genera; (**D**) Archaeal species.

The shotgun dataset contained 20 bacterial phyla, 91 families, and 303 genera. *Firmicutes* (29.77%), *Proteobacteria* (28.38%), *Bacteroidetes* (14.87%), *Actinobacteria* (9.34%) and *Cyanobacteria* (4.81%) were the most abundant phyla. While *Cyanobacteria* are typically native to marine environments, they are routinely reported in studies of the bovine GIT microbiota [2,3,23,24]. Recent studies showed that these taxa likely belong to a distinct *Cyanobacteria-like* lineage, also known as Melainabacteria [23], that exist in the gut of human [25] and termite [26]. The genome features of a recently sequenced *Melainabacteria* indicated its capacity in fermenting sugars and chitobiose and producing H_2_, ethanol, and D-lactate [24]. Further investigation of phylogeny of the rumen *Cyanobacteria* (or *Melainabacteria*) may offer insight into their role in rumen microbial fermentation. *Prevotellaceae* (8.48%), *Clostridiaceae* (7.35%), *Bacillaceae* (2.94%), *Mycoplasmataceae* (2.16%) and *Lactobacillaceae* (1.98%) were the dominant families. The genus-level profile was dominated by *Prevotella* (10.12%), *Clostridium* (8.66%), *Bacillus* (2.87%), *Mycoplasma* (2.42%) and *Fibrobacter* (2.34%) (Figure 1A–C). While the identities of these predominant bacterial taxa were similar to those reported in other metagenomics studies, the relative abundance of Proteobacteria was higher than that commonly reported in beef steers [27,28]. One reason for this discrepancy could be the different approaches applied for taxonomy assignment. While both Brulc et al. [27] and Wallace et al. [28] utilized metagenomic sequencing, microbial classification was still assigned using extracted 16S rRNA gene sequences. In the current study, rRNA genes represent a very small portion of the metagenome (typically <1000 reads per sample), which was not sufficient for taxonomy assignment purposes. Therefore, all reads were aligned to the reference for taxonomy assignment. *Bacillus* was not commonly detected from previous amplicon sequencing-based studies [5,24]. We speculate that its high prevalence in the rumen of bull cattle may be a unique feature compared to other ruminants, and their exact function in the rumen needs further exploration. *Proteobacteria* was reported to be more prevalent in the gut of male than that of female in different host species [29,30,31] as such it may also be true that bull cattle indeed host higher *Proteobacteria* in the rumen compared to cows and steers, but this must be validated.

Shotgun sequencing identified 171 archaeal species in the rumen, exposing a more diverse archaeal community in bull cattle compared to other ruminant animals such as dairy cows [13] and beef steers [28,32] using the same approach. *Methanobrevibacter* sp. YE315 (17.32%), *Methanobrevibacter olleyae* (8.15%), *Methanobrevibacter millerae* (7.95%), *Methanobrevibacter ruminantium* (7.63%), and *Methanococcus voltae* (4.56%) were the most abundant archaeal species (Figure 1D).

Of these five predominate archaea, only *Methanobrevibacter ruminantium* was previously reported to be among the predominant archaeal species of the rumen [28,33,34]. Of the other four species, *Methanobrevibacter* sp. YE315 was isolated from pooled rumen fluid of four *Bos indicus* cross finisher cattle fed with hay diet [35]; *Methanobrevibacter olleyae* has been identified from beef steers by PCR-DGGE [36]; *Methanobrevibacter millerae* was identified in reindeer using 16S rRNA gene cloning assays [37]; and *Methanococcus voltae,* to the best of our knowledge, has not been previously reported from the rumen. The metagenomic approach did not detect *Methanobrevibacter gottschalkii*, one of the predominant methanogenic species in the rumen as reported by many studies [5,38]. Owing to the lack of a sequenced genome of the *Methanobrevibacter gottschalkii* clade in the database used, this species was not detected via shotgun sequencing in the present study. 

As the rumen microbiota is highly specialized, to precisely assign the sequence reads to the rumen-related phylotypes, we used the customized Kraken database which will allow the sequence interpretation more related to rumen. However, there was very limited whole genome sequence available for the rumen protozoa and fungi species in the Kraken database, and we were unable to classify the Eukaryotes reads to lower phylogenic levels. In the future study, the newly sequenced genomes specifically for rumen protozoa and fungi need to be incorporated to the existing database for better resolution for the rumen microbiome profiling.

Overall, microbial profiles in Angus bull cattle as revealed by shotgun sequencing seemed to differ somewhat from those reported in other cattle, suggesting the divergent nutrition requirements of bulls compared to other ruminant animals are accompanied by differences in rumen microbial composition.

### 3.2. Microbial Profiles Generated Using Amplicon Sequencing

Amplicon sequencing of 16S rRNA and 18S rRNA gene fragments generated a total of 16,335 ± 1512 bacterial sequences, which were assigned to 1604 ± 743 bacterial OTUs; 1537 ± 509 archaeal sequences, assigned to 660 ± 267 archaeal OTUs; and 1378 ± 370 protozoal reads, assigned to 1094 ± 411 OTUs for each sample (Table 4). Detailed taxonomy assignment data is provided in File S2.

**Figure 2:**
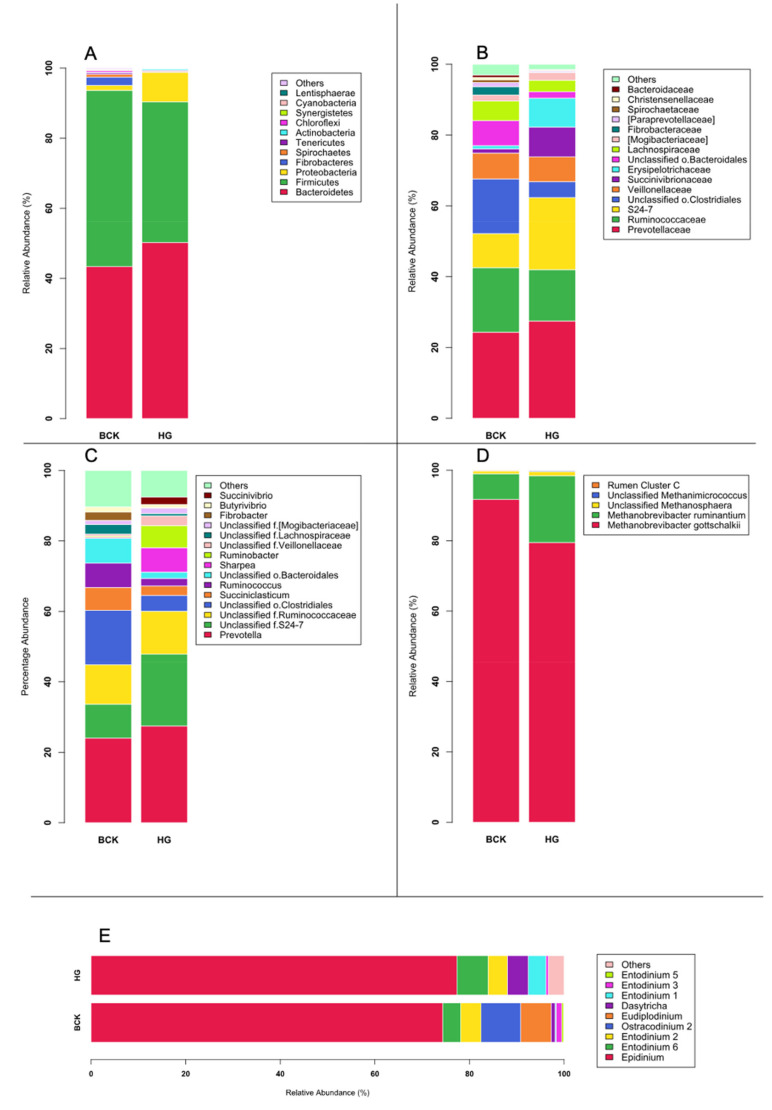
Microbial profiles at different phylogeny levels observed by amplicon-seq. (**A**) Bacterial phyla; (**B**) Bacterial families; (**C**) Bacterial genera; (**D**) Archaeal species; (**E**) Protozoal species.

Eleven bacterial phyla, 28 families, and 48 genera were identified using amplicon sequencing. The most abundant bacterial phyla were *Bacteroidetes* (46.81%), *Firmicutes* (45.20%), *Proteobacteria* (4.92%), *Fibrobacter* (1.22%) and *Spirochaetes* (0.56%). The family level profile was dominated by *Prevotellaceae* (25.91%), *Ruminococcaceae* (16.37%), S24-7 (15.01%), unclassified o.*Clostridiales* (9.97%), and *Veillonellaceae* (7.15%) at family level. *Prevotella* (25.72%), unclassified family (f) *S24*-7 (15.01%), unclassified f.*Ruminococcaceae* (11.69%), unclassified o.*Clostridiales* (9.97%), and *Succiniclasticum* (4.58%) were the dominant genera (Figure 2A–C). The bacterial profiles revealed by amplicon sequencing in this study were similar to those commonly reported in other ruminants using either 454 pyrosequencing [33] or Illumina MiSeq sequencing [10], characterized by the predominance of *Bacteroidetes* and *Firmicutes*, *Prevotellaceae* and *Ruminococcaceae*, and *Prevotella* at the phylum, family and genus levels, respectively. 

Four archaeal species were identified, with *Methanobrevibacter gottschalkii* predominant (85.59%), followed by *Methanbrevibacter ruminantium* (13.14%), *Methanosphaera* sp. (1.00%), and *Thermoplasmatales* spp. (0.14%) (Figure 2D).

Primer bias is an inherent limitation of amplicon sequencing [39]. Using the same primer set as in the present study, *Methanobrevibacter ruminantium* and *Methanobrevibacter gottschalkii* were the dominant archaeal taxa in previous studies [2,40]. However, archaeal diversity is lower in the current study than previously reported [2,40], suggesting that the rumen archaeal community in the rumen of Bulls may be indeed less complicated than that in beef steers/heifers and dairy cows. We are reticent that this may also be caused by the low depth of 454 pyrosequencing compared to Illumina platforms, which may identify fewer rare taxa. However, elevated abundance of *Methanobrevibacter gottschalkii* was reported in high methane-emitting beef and dairy cattle [41], and thus the extremely high proportion of *Methanobrevibacter gottschalkii* in this study may explain why bulls produce more methane than cows, heifers, calves and other cattle [42].

Nine protozoal genera were identified, with *Epidinium* sp. (75.83%) as the most abundant species (Figure 2E). It was reported that small protozoa such as *Entodinium* could be under-represented and large protozoa such as *Epidinium* could be over-represented by pyrosequencing method compared to microscopy [43]. This may explain the observed predominance of *Entodinium* in the current study. Regardless of the potential bias introduced by sequencing method, the proportion of *Epidinium* sp. was still higher than previously reported from ruminants [15]. *Epidinium* are known to harbor a significant number of protozoan-associated methanogens (PAM) [44], and as such it is not surprising to observe its prevalence in the rumen of bull cattle, which produce more methane (per head as determined by emission factor) than other ruminant animals [45]. 

### 3.3. Shotgun-Seq and Amplicon-Seq Revealed Different Rumen Microbial Communities in Bull Cattle

The size of the shotgun sequencing dataset was comparable to previous studies of the rumen metagenome [27,45], with over 20 million reads retrieved from each sample. Rarefaction curves showed a plateau (Figure S1), indicating sufficient depth of sequencing for full coverage of microbial diversity.

As for the data obtained using amplicon sequencing, the Good’s Coverage score in most of the samples was higher than 0.95, and the alpha diversity indices were similar to previous studies using amplicon sequencing [46,47]. Therefore, we concluded that the entire dataset generated in the current study was appropriate for downstream analyses.

**Figure 3:**
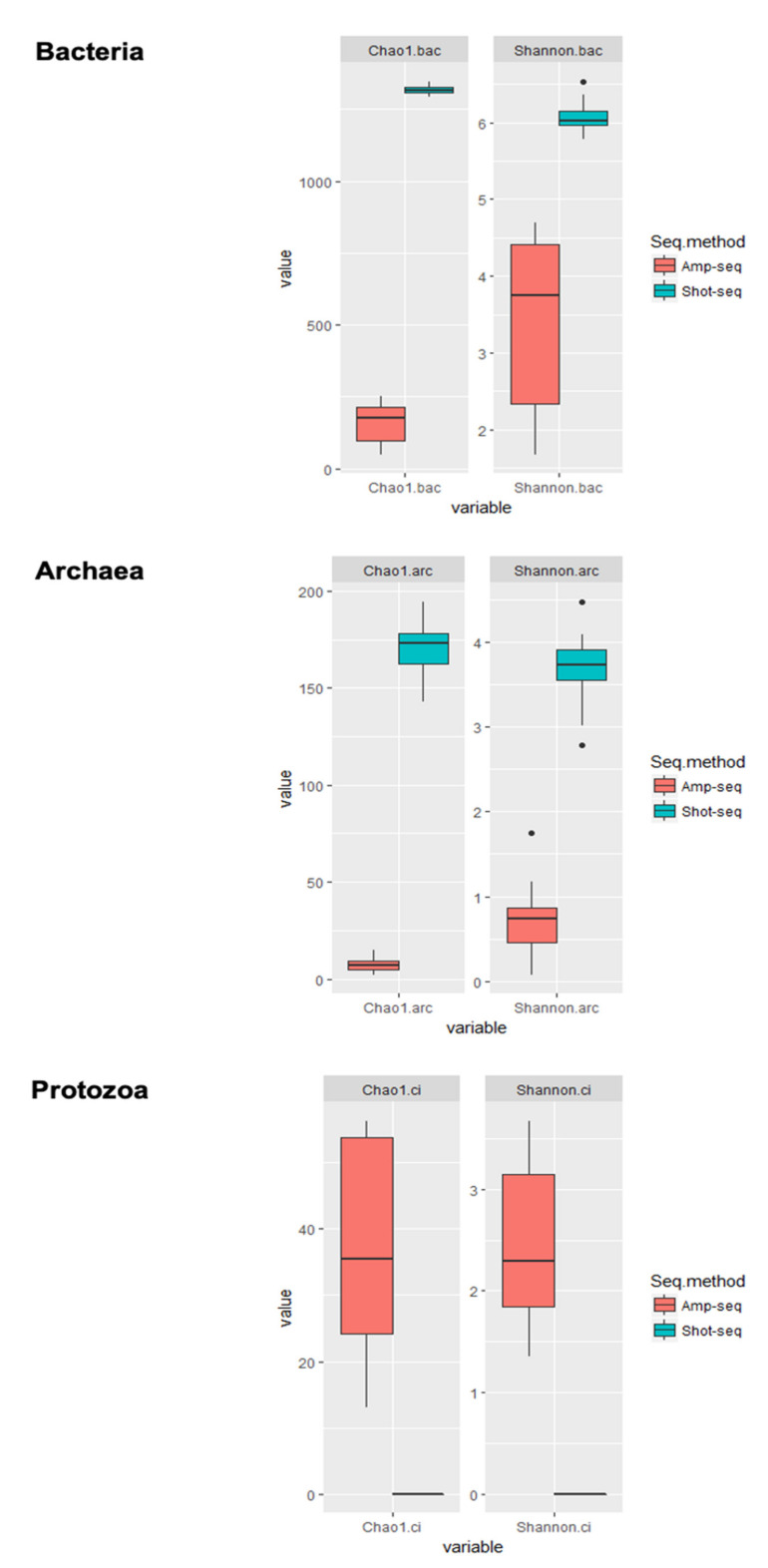
Alpha diversity (Chao 1) and beta diversity (Shannon) indices of bacterial, archaeal, and protozoal communities by shotgun-seq and amplicon-seq.

While both sequencing approaches generated taxonomic profiles, which were generally representative of the microbiome, there were significant differences between both. As shown in Figure 3, more diverse bacterial and archaeal communities were identified using shotgun sequencing, whereas protozoal taxa were only identified using amplicon sequencing.

Moreover, the predominant taxa differed between the two sequencing methods. As two different sequencing platforms were used for amplicon sequencing and shotgun sequencing, such differences may also be due to the method variation. With the longer sequence fragments being generated by amplicon sequencing method, it is expected that the taxonomy assignment was more precise and accurate. Meanwhile, to generate sufficient reads for metagenome analysis, Illumina sequencing allowed higher data output and thus it was chosen to be used for shotgun sequencing. Although the primers used in the amplicon sequencing method were all universal primers which were expected to amplify all taxa, the amplification efficiency of each taxon may be different leading to the discrepancies between these two sequencing methods.

**Figure 4:**
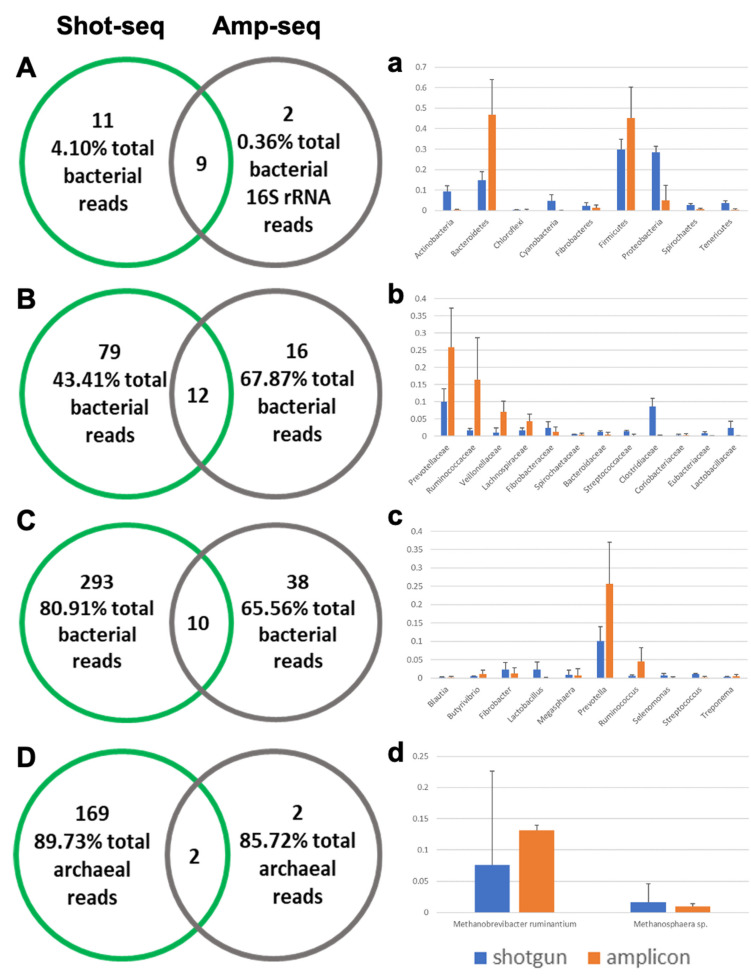
Comparison of the identified phylotypes by shotgun-seq and amplicon-seq. (**A**–**D**) Venn diagrams of the bacterial phyla, bacterial families, bacterial species, and archaeal species. (**a**–**d**) Relative abundance of the shared phylotypes of the bacterial phyla, bacterial families, bacterial species, and archaeal species.

When the taxonomic profiles were compared across sequencing method, 9 bacterial phyla, 12 bacterial families, 10 bacterial genera, and 2 archaeal species were common to both. However, most of the taxa were only detected using one sequencing method (Figure 4A–D).

Even for the shared taxa, their relative abundance differed substantially between both methods (Figure 4a–d). In addition to the greater depth of sequencing in the shotgun dataset, which led to more taxa being identified, this is also likely due to the divergent methods of taxonomic assignment. A locally-built microbial genome reference previously described by Neves et al. [48] was updated for assigning the taxonomy in the shotgun data, which contained the complete microbial genomes from RefSeq (https://www.ncbi.nlm.nih.gov/refseq, accessed on 15 May 2019) and the Hungate1000 project at JGI (https://genome.jgi.doe.gov/TheHunmicrobiome/, accessed on 20 October 2019). Therefore, only the microorganisms with an available whole genome sequence were included in the shotgun dataset in the current study. As such, certain important taxa, like the archaeal species *Methanobrevibacter gottschalkii* and the protozoal genus *Entodinium*, whose genome sequences were not available at the time of database construction, were not included in the reference. Moreover, the database used for analysis of the amplicon sequencing data contains representative sequences of both sequenced and un-sequenced microorganisms, and thus it is not surprising to see different results being revealed by the two methods. Although Ibarbalz et al. [49] claimed that the primer-induced bias associated with amplicon sequencing did not impact the quantitative examination of microbial communities, previous studies which compared amplicon and shotgun sequencing approaches showed only weak correlations [50,51], which was replicated in the present study. 

### 3.4. Dietary Effect on Rumen Microbiota Revealed by Shotgun-Seq and Amplicon-Seq

**Figure 5:**
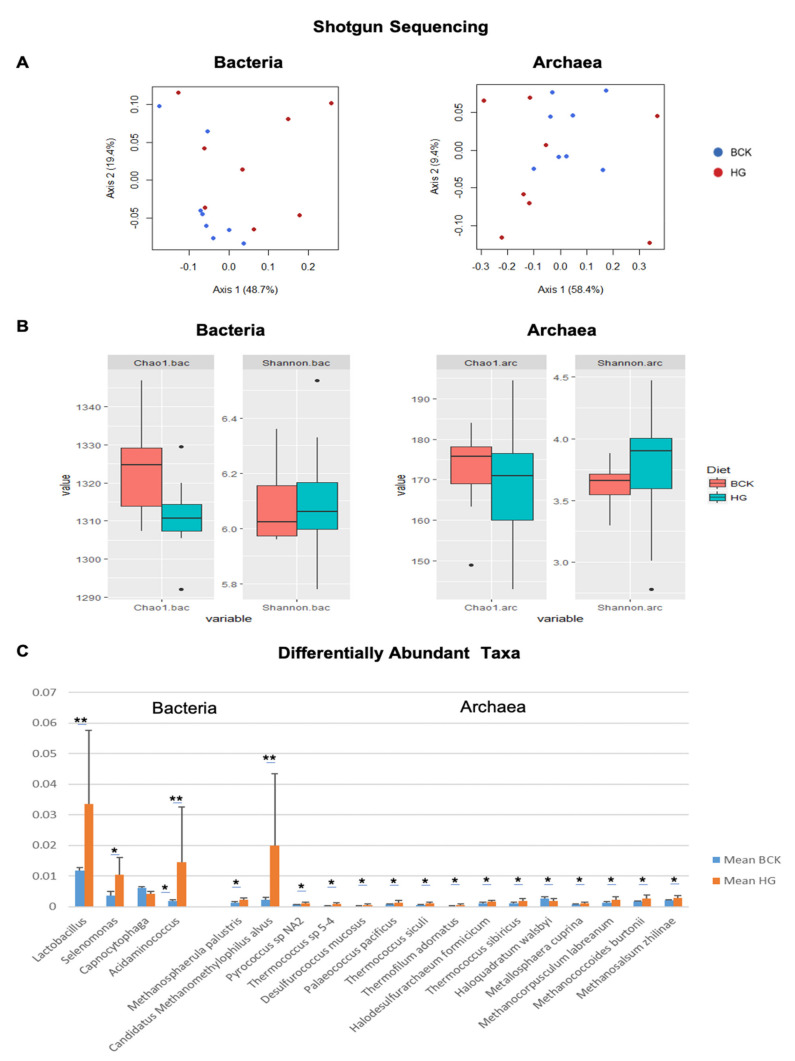
Comparison of microbial profiles observed by shotgun-seq. (**A**) PCoA plot of bacterial and archaeal communities. (**B**) Alpha (Chao 1) and beta (Shannon) indices of bacterial and archaeal communities. (**C**) Differential abundant phylotypes observed between the two diets. Significance was indicated as * fdr < 0.05 and ** fdr < 0.01.

Diet is known to be a key determinant of the rumen microbiota [5] As the two sequencing methods revealed distinctive microbial communities, we assessed the dietary effect on these communities separately, to ascertain if any similar patterns were evident. Given previous reports of diet-based divergence in microbial communities across ruminant species and using different sequencing methods [5,52], it was surprising to see that the shotgun sequencing data did not show a significant dietary effect on microbial structure (ANOSIM > 0.189; Figure 5A) in the present study. Alpha and beta indices of bacterial and archaeal communities were similar across diets (Figure 5B), suggesting that shotgun sequencing may not be the most appropriate method to describe microbial community changes in response to diet. Nevertheless, differentially abundant taxa, including 4 bacterial genera and 15 archaeal species, were still identified between the two diets (Figure 5C) using shotgun sequencing. It is also possible that the greater depth of shotgun sequencing might magnify individual variation between animals, masking some difference between groups. 

**Figure 6:**
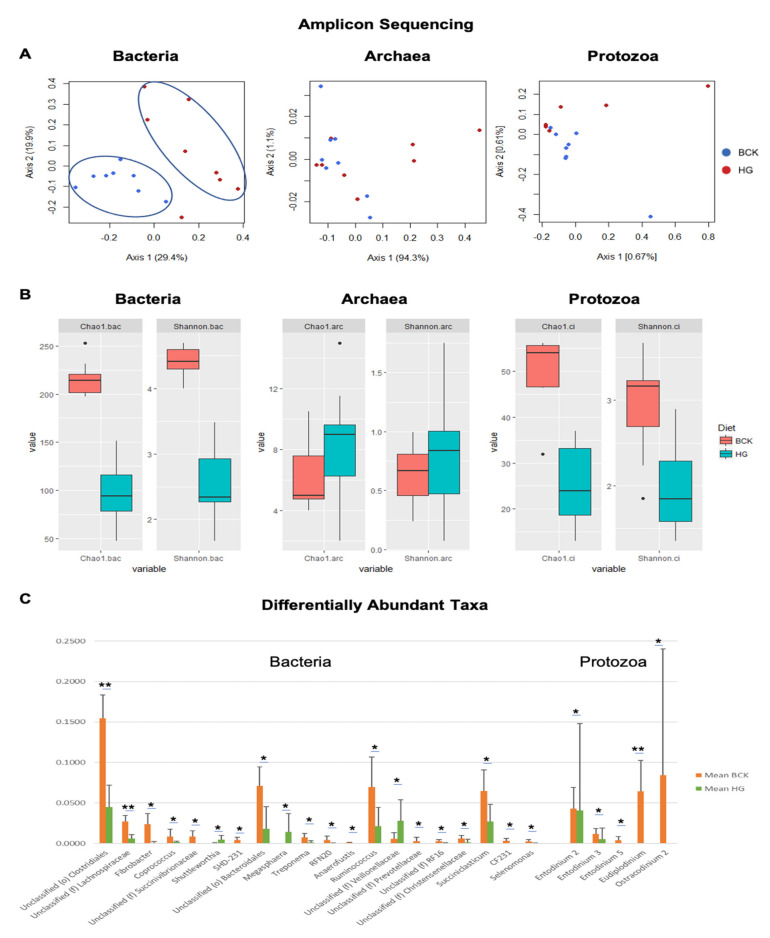
Comparison of microbial profiles observed by shotgun-seq. (**A**) PCoA plot of bacterial, archaeal, and protozoal communities. (**B**) Alpha (Chao 1) and beta (Shannon) indices of bacterial, archaeal, and protozoal communities. (**C**) Differential abundant phylotypes observed between the two diets. Significance was indicated as * fdr < 0.05 and ** fdr < 0.01.

Amplicon sequencing revealed stronger dietary effects on the bacterial profiles (ANOSIM R = 0.434, *p* < 0.001), with clear diet-based clustering evident (Figure 6A). However, archaeal (ANOSIM R = 0.032) and protozoal (ANOSIM R = 0.181) profiles exhibited greater similarity across diets (Figure 5A). As reflected by alpha and beta diversity indices, the bacterial and protozoal communities in bulls fed BCK diet were more diverse than those fed HG diet (*p* < 0.05) (Figure 6B), which agrees with previous findings in other cattle [53]. Seventeen bacterial genera and five protozoal species were more abundant in BCK diet-fed bulls, and three bacterial genera were more abundant in HG diet-fed bulls (Figure 6C).

There were few consistent effects of diet on the microbial community which were identified using both sequencing approaches, and indeed, a number of opposing responses were observed. Previous studies reported that higher abundance of *Firmicutes* is associated with forage-rich diets, while *Bacteroidetes* are predominant in animals fed high-grain diet [54,55]. This broadly agrees with our amplicon sequencing data, whereby the proportion of Firmicutes was numerically higher in BCK animals and Bacteroidetes was numerically higher in HG diet (Figure 2A), though these differences were not statistically significant. However, opposite trends were evident in the shotgun dataset, with higher numbers of Bacteroidetes present in BCK-fed bulls and greater abundance of Firmicutes in HG animals (Figure 1A). Though similar findings were previously reported in plains bison [56], they have not been observed in cattle. Given the divergent results concerning these predominant bacterial phyla using amplicon and shotgun sequencing, further validation to quantify both groups using qPCR is required to determine which sequencing method can provide more accurate assessment of the rumen microbiota in response to dietary changes. 

Similarly, shotgun sequencing showed *Selenomonas* was more abundant under the HG diet (Figure 5C), but this genus was more abundant in BCK diet when assessed using amplicon sequencing (Figure 6C). As the nature of the amplicon-seq approach employed here did not allow accurate taxonomic assignment at the species level, it is not clear whether the *Selenomonas* identified by the two methods contain the same species/OTUs or not. If the *Selenomonas* phylotypes identified from the two methods derive from different species/strains belonging to this genus, they may have different substrate preference. As such, it is not surprising to identify different responses to the two diets with the two sequencing methods. When the major rumen species *Selenomonas ruminantium* was quantified using qPCR, it was reported to be more abundant in the rumen of feedlot steers fed with a high fiber diet in one study [57], but its abundance was higher in the rumen of high-grain diet fed feedlot cattle in another study [58]. This indicates even strain level variation in substrate preference may exist in this genus, and further experiments to quantify *Selenomonas* may help to verify the results from both sequencing methods reported here. 

Irrespective of the taxonomic divergence in dietary effects revealed by both methods, amplicon-seq provided a much more distinct separation of BCK and HG animals in both α- and β-diversity. We cannot conclude if this indicates amplicon sequencing out-performs shotgun-seq in revealing dietary effects, and the less distinct separation across diets in the shotgun-seq dataset may simply be due to individual variation masking the biological differences, due to the greater sequencing depth. 

A major drawback of the current study was that the amplicon data was generated by pyrosequencing, which is out of date and rarely used. However, the longer reads obtained by pyrosequencing technique compared to the commonly used MiSeq are still of value in that it covers longer gene fragment, which allows more accurate assignment of the taxonomy especially the highly correlated phylotypes. The data generated from the current study still provides useful information for sequencing method and platform selection.

As the major objective of the current study was to investigate if comparable results can be generated from the same samples using two different sequencing approaches, our results clearly indicate the necessity to remain reticent of the biases introduced by choice of sequencing approach when interpreting data. This is a major hurdle to the long-term application of studies of the rumen microbiome, but a similar issue has been addressed to an extent in the human gut. *MetaMeta* is a tool developed by Piro et al. [59], aimed at facilitating cross-platform studies of the human gut microbiome. In *MetaMeta*, six metagenomic data processing tools, including CLARK [60], DUDes [61], GOTTCHA [62], KRAKEN [11], KAIJU [63] and mOTUs [64] are embedded. Moreover, different sequencing methods require, by their nature, different database. The integration of a recently published rumen-specific database from the Hungate1000 project [65] would allow more accurate taxonomy assignment with rumen microbial studies, which should be incorporated into the future data analyses pipelines. As suggested by Neves et al. [48], when the rumen microbial reference databases are advanced to a level comparable to that of those of the human gut microbiota, a similar rumen-specific pipeline should provide more complete, reliable, and comparable analyses. 

## 4. Conclusions

Understanding the precise composition of the rumen microbiome in all stages of beef production systems is key in the integration of such data to improve host performance. However, there is little congruence as to the optimal sequencing approach for such studies. Here, we utilized two commonly employed sequencing approaches to characterize the poorly studied rumen microbiome of intact beef bulls. Our data showed that the microbial community revealed by shotgun-seq and amplicon-seq differed significantly. Regardless of variation according to sequencing approach, both methods showed that Angus bulls harbored a different microbial community to those reported in other cattle (e.g., dairy cows, steers) to some extent, indicating that the rumen microbiota in bulls may have a unique structure that reflects their different physiological requirements. Although metagenomic sequencing showed higher resolution in revealing more bacterial and archaeal taxa within the samples, amplicon sequencing outperformed it in revealing the protozoal composition. As we noted very few trends in terms of diet-related differentially abundant taxa across the two methods, we could not conclude which is the optimal approach for characterizing rumen microbial taxonomy. This may be attributable to the divergent approaches to taxonomic assignment, and therefore we propose that a pipeline containing multiple tools and an integrated database suitable for both marker-based and shotgun datasets be developed, to provide better resolution for microbial profiling and facilitating robust comparisons of data generated using different approaches. 

## Figures and Tables

**Table 1:** Ingredient and nutrient composition of the transition diets used to adapt *Bos taurus* bulls to the backgrounding and finishing diets.

Item	Backgrounding (BGK) Diets			High-Grain (HG) Diets							
	BGK1	BGK2	BGK3	HG1	HG2	HG3	HG4	HG5	HG6	HG7	HG8
Inclusion rate, % dry matter (DM)											
Barley Silage	65	55	45	65	55	45	35	25	20	15	10
Barley Grain	25	35	45	25	35	45	55	65	70	75	80
Pellet ^1^	10	10	10	10	10	10	10	10	10	10	10
Days fed	1 to 7	8 to 14	15 till slaughter	1 to 4	5 to 8	9 to 12	13 to 16	17 to 20	21 to 24	24 to 27	28 till slaughter
Nutrient composition, DM basis											
CP	14.0	14.1	14.1	14.0	14.0	14.1	14.2	14.3	14.3	14.4	14.4
NDF	40.9	37.1	33.4	40.9	37.2	33.4	29.7	25.9	24.0	22.1	20.3
Starch	23.2	27.8	32.5	22.9	27.6	32.3	36.9	41.6	43.9	46.3	48.6
Ca	0.8	0.7	0.7	0.8	0.8	0.8	0.7	0.7	0.7	0.7	0.6
P	0.4	0.4	0.4	0.4	0.4	0.4	0.4	0.4	0.4	0.4	0.4

^1^ Contained (% DM) ground barley grain (50.5), corn distillers grain with solubles (25), limestone (8.7), dynamate (6.8), canola meal (5.9), salt (2.1) and a trace mineral and vitamin pre-mix (1). The pellet contained (% DM) CP (13.6), crude fat (3.4), Salt (2.30), Ca (3.44), P (0.54), Mg (1.11), K (1.78), S (1.81), microminerals (mg/kg) Co (4.6), Cu (146.1), I (8.0), Fe (451.8), Mn (335.4), Se (2.26), Zn (313.7), Fl (11.3), and vitamins (IU/kg) A (40,000), D3 (15,000), and E (300).

**Table 2:** Body weight and dry matter intake for *Bos taurus* fed either a backgrounding or finishing diet.

Item ^1^	Backgrounding	Finishing
Initial BW, kg	422	419
Start of test BW, kg	454	453
Ending BW, kg	536	558
DMI, kg/d	13.4	13.2

^1^ Data from the final week of feeding were used for statistical analysis. This equated to week (wk) 7 for backgrounding and wk 9 for finishing. End of test body weight was used to calculate DMI as a percent of BW.

**Table 3:** Alpha and beta diversity of each sample by Shotgun-seq.

Microbe	Sample	Diet	Assembled Contigs	Kraken Aligned Reads	Chao1	Shannon
Archaea	105	HG	164,912	4587	171.50	3.97
	107	HG	281,557	9368	194.50	3.84
	131	HG	332,247	7625	170.32	4.08
	147	HG	86,704	4188	142.97	3.01
	155	HG	114,590	1965	174.89	4.47
	217	BCK	254,337	8301	175.12	3.59
	227	HG	325,493	13,375	159.53	2.78
	23	HG	251,609	5124	181.21	3.98
	273	BCK	355,723	8500	170.97	3.88
	297	HG	341,391	9367	160.18	3.79
	343	BCK	448,664	13,913	177.81	3.70
	381Y	BCK	451,291	11,885	163.34	3.70
	61	BCK	350,872	14,375	148.97	3.62
	81	BCK	214,350	6499	178.84	3.75
	865	BCK	335,964	9230	176.43	3.40
	87	BCK	296,022	9432	184.03	3.30
Bacteria	105	HG	164,912	73,165	1310.67	6.09
	107	HG	281,557	141,287	1312.52	6.11
	131	HG	332,247	164,026	1305.38	6.03
	147	HG	86,704	35,030	1292.00	6.02
	155	HG	114,590	46,849	1310.63	6.33
	217	BCK	254,337	105,263	1329.69	5.96
	227	HG	325,493	111,886	1320.00	6.54
	23	HG	251,609	123,501	1308.09	5.78
	273	BCK	355,723	141,330	1323.82	6.13
	297	HG	341,391	145,019	1329.56	5.92
	343	BCK	448,664	190,508	1325.74	6.02
	381Y	BCK	451,291	172,049	1347.00	6.23
	61	BCK	350,872	182,443	1307.35	6.03
	81	BCK	214,350	87,436	1307.86	6.36
	865	BCK	335,964	142,313	1315.88	5.98
	87	BCK	296,022	133,750	1329.02	5.96

**Table 4:** Alpha and beta diversity of each sample by Amplicon-seq.

Microbe	Sample	Diet	Seqs	Chao1	Goods Coverage	Shannon
Archaea	105	HG	1453	7.00	0.99	0.95
	107	HG	1764	9.00	0.99	0.44
	131	HG	2198	15.00	0.97	0.84
	147	HG	2261	9.00	1.00	1.18
	155	HG	2661	11.50	0.98	1.75
	217	BCK	1233	9.33	0.99	1.00
	227	HG	1659	4.00	0.98	0.83
	23	HG	1459	2.00	1.00	0.07
	273	BCK	1282	10.50	0.96	0.80
	297	HG	1500	9.00	0.99	0.49
	343	BCK	1467	4.00	0.98	0.47
	381Y	BCK	1192	5.00	0.99	0.43
	61	BCK	1239	5.00	0.98	0.83
	81	BCK	1434	4.00	0.99	0.24
	865	BCK	462	7.00	0.97	0.66
	87	BCK	1323	5.00	0.99	0.67
Bacteria	105	HG	16,378	100.83	0.95	2.31
	107	HG	14,037	87.30	0.96	2.34
	131	HG	16,059	144.88	0.93	3.07
	147	HG	15,974	47.43	0.97	1.67
	155	HG	17,090	63.14	0.98	2.34
	217	BCK	18,187	231.29	0.85	4.38
	227	HG	16,327	151.25	0.90	3.48
	23	HG	16,869	83.40	0.96	2.17
	273	BCK	15,886	215.33	0.87	4.00
	297	HG	13,406	106.55	0.93	2.88
	343	BCK	15,412	212.33	0.84	4.66
	381Y	BCK	16,456	217.23	0.86	4.43
	61	BCK	18,355	253.09	0.85	4.70
	81	BCK	19,457	197.25	0.86	4.58
	865	BCK	15,326	201.00	0.82	4.40
	87	BCK	16,137	201.68	0.87	4.07
Protozoa	105	HG	1006	17.75	0.98	1.35
	107	HG	721	37.00	0.97	2.28
	131	HG	1424	34.00	0.96	1.81
	147	HG	1441	19.00	0.99	2.31
	155	HG	1820	33.00	0.96	1.62
	217	BCK	1645	55.43	0.94	2.84
	227	HG	779	13.00	1.00	2.90
	23	HG	1222	24.33	0.97	1.47
	273	BCK	1827	32.00	0.96	1.85
	297	HG	1434	23.50	0.98	1.88
	343	BCK	1537	46.67	0.95	3.19
	381Y	BCK	1039	56.14	0.94	3.13
	61	BCK	1808	53.36	0.94	3.21
	81	BCK	1357	54.60	0.94	3.29
	865	BCK	1128	46.38	0.95	3.67
	87	BCK	1862	56.20	0.94	2.24

## Data Availability

All sequences supported the results of the current manuscript have been submitted to NCBI. The amplicon-based data is available under project ID PRJNA438952. Metagenomic data can be accessed via project ID PRJNA438535.

## Supplementary Materials

The following are available online at https://www.mdpi.com/article/10.3390/vetsci8070138/s1, Figure S1: Rarefaction curve of the samples, File S1: PCR conditions, File S2: Taxonomy assignment for individual samples.

## References

[1] Guan L.L., Nkrumah J.D., Basarab J.A., Moore S.S. (2008). Linkage of microbial ecology to phenotype: Correlation of rumen microbial ecology to cattle’s feed efficiency. FEMS Microbiol. Lett..

[2] Li F., Guan L.L. (2017). Metatranscriptomic Profiling Reveals Linkages between the Active Rumen Microbiome and Feed Efficiency in Beef Cattle. Appl. Environ. Microbiol..

[3] McGovern E., Kenny D.A., McCabe M.S., Fitzsimons C., McGee M., Kelly A.K., Waters S.M. (2018). 16S rRNA Sequencing Reveals Relationship Between Potent Cellulolytic Genera and Feed Efficiency in the Rumen of Bulls. Front. Microbiol..

[4] Jami E., Whit B.A., Mizrahi I. (2014). Potential role of the bovine rumen microbiome in modulating milk composition and feed efficiency. PLoS ONE.

[5] Henderson G., Cox F., Ganesh S., Jonker A., Young W., Collaborators G.R.C., Janssen P.H. (2015). Rumen microbial community composition varies with diet and host, but a core microbiome is found across a wide geographical range. Sci. Report.

[6] Svartstrom O., Alneberg J., Terrapon N., Lombard V., de Bruijn I., Malmsten J., Dalin A.-M., Muller E.E.L., Shah P., Wilmes P. (2017). Ninety-nine *de novo* assembled genomes from the moose (*Alces alces*) rumen microbiome provide new insights into microbial plant biomass degradation. ISME J..

[7] Stewart R.D., Auffret M.D., Warr A., Wiser A.H., Press M.O., Langford K.W., Liachko I., Snelling T.J., Dewhurst R.J., Walker A.W. (2018). Assembly of 913 microbial genomes from metagenomic sequencing of the cow rumen. Nat. Commun..

[8] De Santis T.Z., Hugenholtz P., Larsen N., Rojas M., Brodie E.L., Keller K.T., Huber T., Dalevi D., Hu P., Andersen G.L. (2006). Greengenes, a chimera-checked 16S rRNA gene database and workbench compatible with ARB. Appl. Environ. Microbiol..

[9] Quast C., Pruesse E., Yilmaz P., Gerken J., Schweer T., Yarza P., Peplies J., Glöckner F.O. (2013). The SILVA ribosomal RNA gene database project: Improved data processing and web-based tools. Nucl. Acids Res..

[10] Myer P.R., Kim M., Freetly H.C., Smith R.P. (2016). Evaluation of 16S rRNA amplicon sequencing using two next-generation sequencing technologies for phylogenetic analysis of the rumen bacterial community in steers. J. Microbiol. Methods.

[11] Wood D.E., Salzberg S.L. (2014). Kraken: Ultrafast metagenomic sequence classification using exact alignments. Genome Biol..

[12] Roehe R., DewHurst R.J., Cuthie C.-A., Rooke J.A., McKain N., Ross D.W., Hyslop J.J., Waterhouse A., Freeman T.C., Watson M. (2016). Bovine host genetic variation influences rumen microbial methane production with best selection criterion for low methane emitting and efficiently feed converting hosts based on metagenomic gene abundance. PLoS Genet..

[13] Hess M., Sczyrba A., Egan R., Kim T.-W., Chokhawala H., Schroth G., Luo S., Clark D.S., Chen F., Zhang T. (2011). Metagenomic Discovery of Biomass-Degrading Genes and Genomes from Cow Rumen. Science.

[14] Zhou M., Hernandez-Sanabria E., Guan L.L. (2009). Assessment of the microbial ecology of ruminal methanogens in cattle with different feed efficiencies. Appl. Environ. Microbiol..

[15] Tymensen L., Barkley C., McAllister T.A. (2012). Relative diversity and community structure analysis of rumen protozoa according to T-RFLP and microscopic methods. J. Microbiol. Methods.

[16] Marume U., Kusina N.T., Hamudikuwanda H., Ndengu M., Nyoni O. (2011). Effect of dry season nutritional supplementation on fertility in bulls in Sanyati smallholder farming area, Zimbabwe. Afr. J. Agri. Res..

[17] Bushnell B. (2014). BBMap short read aligner, and other bioinformatic tools. https://sourceforge.net/projects/bbmap/.

[18] Caporaso J.G., Kuczynski J., Stombaugh J., Bittinger K., Bushman F., Costello E.K., Fierer N., Peña A.G., Goodrich J.K., Gordon J.I. (2010). QIIME allows analysis of high-throughput community sequencing data. Nat. Methods.

[19] Edgar R.C. (2010). Search and clustering orders of magnitude faster than BLAST. Bioinformatics.

[20] Li D., Liu C.M., Luo R., Sadakane K., Lam T.W. (2015). MEGAHIT: An ultra-fast single-node solution for large and complex metagenomics assembly via succinct de Bruijn graph. Bioinformatics.

[21] McMurdie P.J., Holmes S. (2013). Phyloseq: An R package for reproducible interactive analysis and graphics of microbiome census data. PLoS ONE.

[22] Clark K.R. (1993). Non-parametric multivariate analysis of changes in community structure. Aust. J. Ecol..

[23] Scharen M., Drong C., Kiri K., Riede S., Gardener M., Meyer U., Hummel J., Urich T., Breves G., Danicke S. (2017). Differential effects of monensin and a blend of essential oils on rumen microbiota composition of transition dairy cows. J. Dairy Sci..

[24] O’Hara E., Kelly A., McCabe M.S., Kenny D.A., Guan L.L., Waters S.M. (2018). Effect of a butyrate-fortified milk replacer on gastrointestinal microbiota and products of fermentation in artificially reared dairy calves at weaning. Sci. Rep..

[25] Soo R.M., Skennerton C.T., Sekiguchi Y., Imelfort M., Paech S.J., Dennis P.G., Steen J.A., Parks D.H., Tyson G.W., Hugenholtz P. (2014). An Expanded Genomic Representation of the Phylum Cyanobacteria. Genome Biol. Evol..

[26] Utami Y.D., Kuwahara H., Murakami T., Morikawa T., Sugaya K., Kihara K., Yuki M., Lo N., Deevong P., Hasin S. (2018). Phylogenetic Diversity and Single-Cell Genome Analysis of “*Melainabacteria*”, a Non-Photosynthetic Cyanobacterial Group, in the Termite Gut. Microbes Environ..

[27] Brulc J.M., Antonopoulos D.A., Miller M.E.B., Wilson M.K., Yannarell A.C., Dinsdale E.A., Edwards R.E., Frank E.D., Emerson J.B., Wacklin P. (2009). Gene-centric metagenomics of the fiber-adherent bovine rumen microbiome reveals forage specific glycoside hydrolases. Proc. Natl. Acad. Sci. USA.

[28] Wallace R.J., Rooke J.A., McKain N., Duthie C.-A., Hyslop J.J., Ross D.W., Waterhouse A., Watson M., Roehe R. (2015). The rumen microbial metagenome associated with high methane production in cattle. BMC Genom..

[29] Li M., Wang B., Zhang M., Rantalainen M., Wang S., Zhou H., Zhang Y., Shen J., Pang X., Wei H. (2008). Symbiotic gut microbes modulate human metabolic phenotypes. Proc. Natl. Acad. Sci. USA.

[30] Kaliannan K., Robertson R.C., Murphy K., Stanton C., Kang C., Wang B., Hao L., Bhan A.K., Kang J.X. (2018). Estrogen-mediated gut microbiome alterations influence sexual dimorphism in metabolic syndrome in mice. Microbiome.

[31] Wu J.F., Muthusamy A., Al-Ghalith G.A., Knights D., Guo B., Wu B., Remmel R.P., Schladt D.P., Alegre M.-L., Oetting W.S. (2018). Urinary microbiome associated with chronic allograft dysfunction in kidney transplant recipients. Clin. Transplant..

[32] Auffret M.D., Stewart R., Dewhurst R.J., Duthie C.-A., Rooke J.A., Wallace R.J., Freeman T.C., Snelling T.J., Watson M., Roeche R. (2017). Identification, comparison, and validation of robust rumen microbial biomarkers for methane emissions using diverse *Bos taurus* breeds and basal diets. Front. Microbiol..

[33] Danman S.E., Fernandea G.M., Shinkai T., Mitsumori M., McSweeney C.S. (2015). Metagenomic analysis of the rumen microbial community following inhibition of methane formation by a halogenated methane analog. Front. Microbiol..

[34] Popova M., McGovern E., McCabe M.S., Martin C., Doreau M., Arbre M., Meale S., Morgavi D., Waters S.M. (2017). The Structural and Functional Capacity of Ruminal and Cecal Microbiota in Growing Cattle Was Unaffected by Dietary Supplementation of Linseed Oil and Nitrate. Front. Microbiol..

[35] Gilbert R., Ouwerkerk D., Klieve A. (2011). Archaeaphage Therapy to Control Rumen Methanogens. Meat and Livestock Australia Final Project Report (B.CCH.1007). http://www.mla.com.au/download/finalreports?itemId=1593.

[36] Zhou M., Hernandez-Sanabria E., Guan L.L. (2010). Characterization of variation in rumen methanogenic communities under different dietary and host feed efficiency conditions, as determined by PCR-denaturing gradient gel electrophoresis analysis. Appl. Environ. Microbiol..

[37] Sundset M.A., Edwards J.E., Cheng Y.F., Senosiain R.S., Fraile M.N., Northwood K., Praesteng K.E., Glad T., Mathiesen S.D., Wright A.-D.G. (2009). Rumen microbial diversity in Svalbard reindeer, with particular emphasis on methanogenic archaea. FEMS Microbiol. Ecol..

[38] Iqbal M.W., Zhang Q., Yang Y., Li L., Zou C., Huang C., Lin B. (2018). Comparative study of rumen fermentation and microbial community differences between water buffalo and Jersey cows under similar feeding conditions. J. Appl. Anim. Res..

[39] Rausch P., Rühlemann M., Hermes B.M., Doms S., Dagan T., Dierking K., Domin H., Fraune S., Von Frieling J., Hentschel U. (2019). Comparative analysis of amplicon and metagenomic sequencing methods reveals key features in the evolution of animal metaorganisms. Microbiome.

[40] Zhou M., Hunerberg M., Chen Y., Reuter T., McAllister T.A., Evans F., Critchley A.T., Guan L.L. (2018). Air-dried brown seaweed, *Ascophyllum nodosum*, alters rumen microbiome in a manner that changes rumen fermentation profiles and lowers the prevalence of foodborne pathgens. mSphere.

[41] Danielsson R., Dicksved J., Sun L., Gonda H., Müller B., Schnürer A., Bertilsson J. (2017). Methane production in dairy cows correlates with rumen methanogenic and bacterial community structure. Front. Microbial..

[42] Rolfe J. (2001). Economics of reducing methane emissions from cattle production in central Queensland. Project Final Report for Meat and Livestock Australia Lid.

[43] Kittelmann S., Devente S.R., Kirk M.R., Seedorf H., Dehority B.A., Janssen P.H. (2015). Phylogeny of the intestinal ciliates including first sequences from Charonina ventriculi and comparison of microscopy and 18S rRNA gene pyrosequencing for rumen ciliate community structure analysis. Appl. Environ. Microbiol..

[44] Ng F., Kittelmann S., Patchett M.L., Attwood G.T., Janssen P.H., Rakonjac J., Gagic D. (2016). An adhesin from hydrogen-utilizing rumen methanogenMethanobrevibacter ruminantium M1 binds a broad range of hydrogen-producing microorganisms. Environ. Microbiol..

[45] Ominski K.H., Boadi D.A., Wittenherg K.M., Fulawka D.L., Basarab J.A. (2007). Estimates of enteric methane emissions from cattle in Canada using the IPCC Tier-2 methodology. Can. J. Anim. Sci..

[46] Pope P.B., Mackenzie A.K., Gregor I., Smith W., Sundset M.A., McHardy A.C., Morrison M., Eijsink V.G.H. (2012). Metagenomics of the Svalbard reindeer rumen microbiome reveals abundance of polysaccharide utilization loci. PLoS ONE.

[47] Kamke J., Kittlemann S., Sino P., Li Y., Tavendale M., Ganesh S., Janssen P.H., Shi W., Froula J., Rubin E.M. (2016). Rumen metagenome and metatranscriptome analyses of low methane yield sheep reveals a *Sharpea*-enriched microbiome characterized by lactic acid formation and utilization. Microbiome.

[48] Singh K., Reddy B., Patel A., Panchasara H., Parmar N., Shah T., Bhatt V., Joshi C., Singh K., Reddy B. (2014). Metagenomic analysis of buffalo rumen microbiome: Effect of roughage diet on Dormancy and Sporulation genes. Meta Gene.

[49] Neves A.L.A., Li F., Ghoshal B., McAllister T., Guan L.L. (2017). Enhancing the resolution of rumen microbial classification from metatranscriptomic data using Kraken and Mothur. Front. Microbiol..

[50] Ibarbalz F.M., Perez M.V., Figuerola E.L.M., Erijman L. (2014). The bias associated with amplicon sequencing does not affect the quantitative assessment of bacterial community dynamics. PLoS ONE..

[51] Jovel J., Patterson J., Wang W., Hotte N., O’Keefe S., Mitchel T., Perry T., Kao D., Mason A., Madsen K.L. (2016). Characterization of the Gut Microbiome Using 16S or Shotgun Metagenomics. Front. Microbiol..

[52] Tessler M., Neumann J.S., Afshinnekoo E., Pineda M., Hersch R., Velho L.F.M., Segovia B.T., Lansac-Toha F.A., Lemke M., DeSalle R. (2017). Large-scale differences in microbial biodiversity discovery between 16S amplicon and shotgun sequencing. Sci. Rep..

[53] McCann J.C., Wiley L.M., Forbes T.D., Rouquette F.M., Tedeschi L.O. (2014). Relationship between the rumen microbiome and residual feed intake-efficiency of Brahman bulls stocked on Bermudagrass pastures. PLoS ONE.

[54] Thomas F.A., Webb M., Ghimire S., Blair A., Olson K., Fenske G.J., Fonder A., Christopher-Hennings J., Brake D., Scaria J. (2017). Metagenomic characterization of the effect of feed additives on the gut microbiome and antibiotic resistome of feedlot cattle. Sci. Rep..

[55] Fernando S.C., Purvis H.T., Najar F.Z., Sukharnikov L.O., Krehbiel C.R., Nagaraja T.G. (2010). Rumen microbial population dynamics during adaptation to a high-grain diet. Appl. Environ. Microbiol..

[56] Pitta D.W., Parmar N., Patel A.K., Indugu N., Kumar S., Prajapathi K.B., Patel A.B., Reddy B., Joshi C. (2014). Bacterial Diversity Dynamics Associated with Different Diets and Different Primer Pairs in the Rumen of Kankrej Cattle. PLoS ONE.

[57] Bergmann G.T. (2017). Microbial community composition along the digestive tract in forage- and grain-fed bison. BMC Vet. Res..

[58] Messana J.D., Carvalho A.L.E.G.F., Ribeiro A.F., Fiorentini G., Castagnino P.D.S., Granja-Salcedo Y.T., Pires A.V., Berchielli T. (2016). Effects of different sources of forage in high-concentrate diets on fermentation parameters, ruminal biohydrogenation and microbiota in Nellore feedlot steers. J. Agric. Sci..

[59] Piro V.C., Matschkowski M., Renard B.Y. (2017). MetaMeta: Integrating metagenome analysis tools to improve taxonomic profiling. Microbiome.

[60] Ounit R., Wanamaker S., Close T.J., Lonardi S. (2015). CLARK: Fast and accurate classification of metagenomic and genomic sequences using discriminative k-mers. BMC Genom。.

[61] Piro V.C., Lindner M.S., and Renard B.Y. (2016). DUDes: A top-down taxonomic profiler for metagenomics. Bioinformatics.

[62] Freitas T.A.K., Li P.-E., Scholz M.B., Chain P.S.G. (2015). Accurate read-based metagenome characterization using a hierarchical suite of unique signatures. Nucleic Acids Res..

[63] Menzel P., Ng K.L., Krogh A. (2016). Fast and sensitive taxonomic classification for metagenomics with Kaiju. Nat. Commun..

[64] Sunagawa S., Mende D.R., Zeller G., Izquierdo-Carrasco F., Berger S.A., Kultima J.R., Coelho L.P., Arumugam M., Tap J., Nielsen H.B. (2013). Metagenomic species profiling using universal phylogenetic marker genes. Nat. Methods.

[65] Seshadri R., Leahy S.C., Attwood G.T., Teh K.H., Lambie S.C., Cookson A.L., Eloe-Fadrosh E.A., Pavlopoulos G., Hadjithomas M., Varghese N. (2018). Cultivation and sequencing of rumen microbiome members from the Hungate1000 Collection. Nat. Biotechnol..

