# Supplementary figures and images for: Accessing Dietary Effects on the Rumen Microbiome: Different Sequencing Methods Tell Different Stories
Source: Vet Sci. 2021 Jul 19;8(7):138. doi: 10.3390/vetsci8070138 (PMC8310016; doi:10.3390/vetsci8070138)

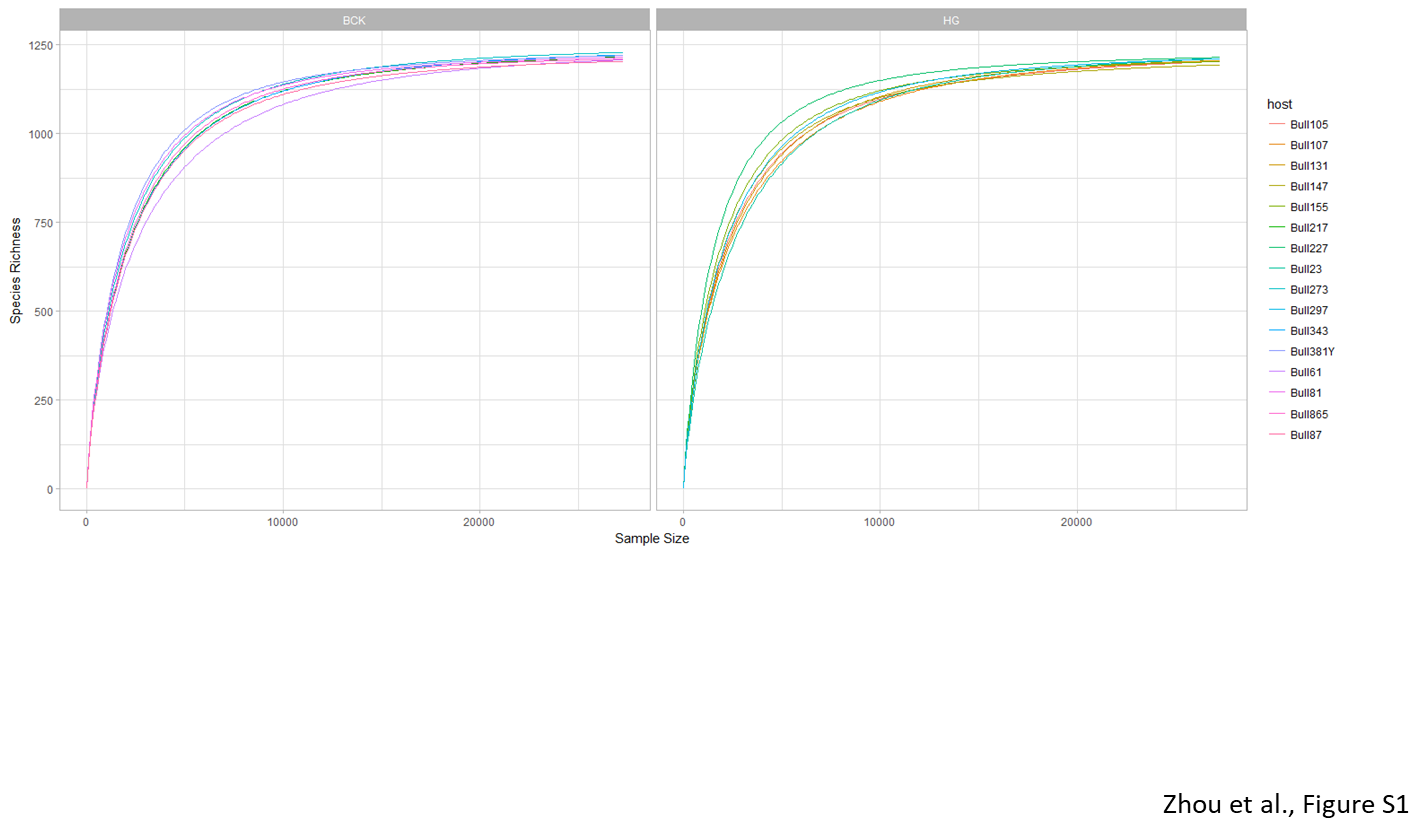

Supplement: Supplementary file 1 [file vetsci-08-00138-s001.zip › R1/ZHOU Vet Sciences R1 FigS1.TIFF]
